# Differences in Connection Strength between Mental Symptoms Might Be Explained by Differences in Variance: Reanalysis of Network Data Did Not Confirm Staging

**DOI:** 10.1371/journal.pone.0155205

**Published:** 2016-11-23

**Authors:** Berend Terluin, Michiel R. de Boer, Henrica C. W. de Vet

**Affiliations:** 1 Department of General Practice and Elderly Care Medicine, EMGO Institute for Health and Care Research, VU University Medical Center, Amsterdam, The Netherlands; 2 Department of Health Sciences and the EMGO Institute for Health and Care Research, Faculty of Earth and Life Sciences, VU University Amsterdam, The Netherlands; 3 Department of Epidemiology & Biostatistics, EMGO Institute for Health and Care Research, VU University Medical Center, Amsterdam, The Netherlands; Katholieke Universiteit Leuven, BELGIUM

## Abstract

**Background:**

The network approach to psychopathology conceives mental disorders as sets of symptoms causally impacting on each other. The strengths of the connections between symptoms are key elements in the description of those symptom networks. Typically, the connections are analysed as linear associations (i.e., correlations or regression coefficients). However, there is insufficient awareness of the fact that differences in variance may account for differences in connection strength. Differences in variance frequently occur when subgroups are based on skewed data. An illustrative example is a study published in PLoS One (2013;8(3):e59559) that aimed to test the hypothesis that the development of psychopathology through “staging” was characterized by increasing connection strength between mental states. Three mental states (negative affect, positive affect, and paranoia) were studied in severity subgroups of a general population sample. The connection strength was found to increase with increasing severity in six of nine models. However, the method used (linear mixed modelling) is not suitable for skewed data.

**Methods:**

We reanalysed the data using inverse Gaussian generalized linear mixed modelling, a method suited for positively skewed data (such as symptoms in the general population).

**Results:**

The distribution of positive affect was normal, but the distributions of negative affect and paranoia were heavily skewed. The variance of the skewed variables increased with increasing severity. Reanalysis of the data did not confirm increasing connection strength, except for one of nine models.

**Conclusions:**

Reanalysis of the data did not provide convincing evidence in support of staging as characterized by increasing connection strength between mental states. Network researchers should be aware that differences in connection strength between symptoms may be caused by differences in variances, in which case they should not be interpreted as differences in impact of one symptom on another symptom.

## Introduction

**Fig 1:**
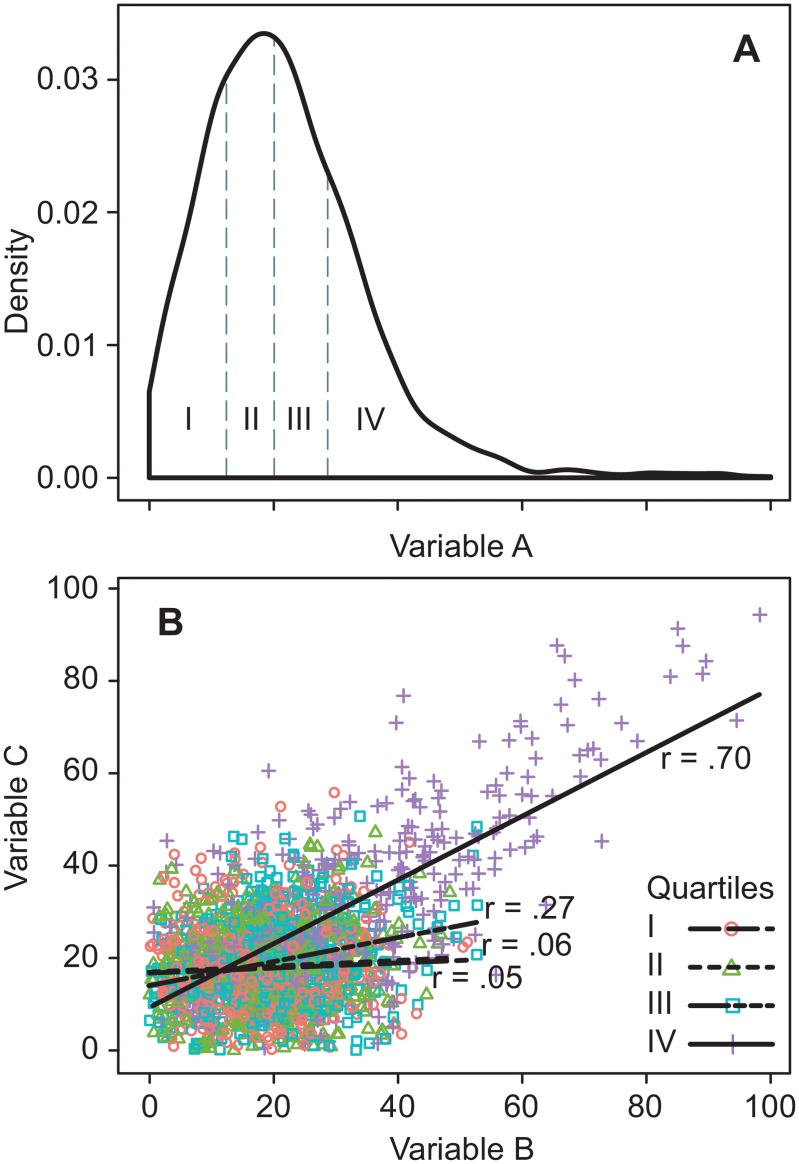
Differential correlations due to different subgroup variances. (A) Density plot of a skewed variable (symptom A) partitioned into 4 quartile groups, demonstrating differential variances across the subgroups. (B) Correlations between 2 skewed variables (symptoms B and C) that are correlated with symptom A and with each other. Across subgroups the correlations vary as a function of different subgroup variances. Subgroup specific correlation coefficients are shown.

The “network approach” to psychopathology conceptualizes mental disorders not as disorders in the organism producing mental symptoms, but merely as symptoms that causally impact on each other [1–3]. Over the past few years, the network approach enjoys growing interest from researchers. The causal connections between symptoms (e.g., between disturbed sleep and fatigue) constitute the networks’ building blocks and differences in “connection strength” are often given crucial significance. However, there is insufficient awareness of a specific pitfall regarding the interpretation of differences in connection strength. The strength of the connection between 2 variables (expressed as correlation or regression coefficient) rests principally on the amount of common (or shared) variance, relative to the total variance (i.e., common variance and unique variance including measurement error). However, the direct or indirect restriction of the variance of one or both variables (“range restriction”) reduces the connection strength [4]. The comparison of (sub)groups with different severity levels may result in different connection strengths between symptoms solely due to differences in variances [5]. Differential connection strength due to differences in variance is particularly a problem when psychological symptoms are studied in relatively healthy samples. As generally the distribution of symptom scores in such samples is positively skewed, dividing the sample into subgroups based on, for example, median or quartile scores results in different variances across the subgroups, with the largest variance in the most severe subgroup. Fig 1A illustrates the positively skewed distribution of symptom A and how subgrouping based on the quartile scores of A leads to subgroups with different variances of A. If symptoms B and C are also positively skewed, and correlated with symptom A and with each other (as psychological symptoms usually do), the variance imbalance across the subgroups may also be observed in symptoms B and C. This may easily produce differential range restriction and, hence, differential connection strength across the subgroups (Fig 1B). Whereas this methodological fallacy is lurking in many network studies (e.g., [3,6,7]), it is particularly salient in a recent study by Wigman et al. [8].

Wigman et al. aimed to test the hypothesis that mental states dynamically impact on each other over time in ways that suggest the development of psychopathology through “staging” (and “profiling”, but we will exclusively focus on staging). They hypothesized that staging was characterized by progressively increasing connection strength between mental states. Wigman et al. tested this hypothesis by analysing Experience Sampling Methodology (ESM) [9] data from a sample of female general population twins. The study participants recorded information on 3 mental states (negative affect, positive affect and paranoia). The sample was divided into 4 severity subgroups, based on the quartile scores of the Symptom Checklist-90-R (SCL) total score, representing different stages of psychopathology. Staging was examined by performing multilevel (levels: measurements within persons within families) linear regression analysis predicting each of the mental states at time *t* by all mental states at the preceding measurement moment time *t-1* and including SCL-severity as an effect modifier in these models. Any of the 3 mental states at time *t* was regressed on any of the 3 mental states at time *t-1*, yielding 9 regression models. Increasing (fixed) regression coefficients with increasing severity, combined with statistical significance of the mental state-severity interaction term, was interpreted as proof of staging. Staging was observed in 6 models in which negative affect (NA) or paranoia (PAR) was the dependent variable. No evidence of staging was found in 3 models in which positive affect (PA) was the dependent variable. Wigman et al. concluded “that more severe stages of psychopathology are characterized by stronger … inter- and intra-mental state connections over time”.

We suspected that differences in the magnitude of the fixed regression coefficients across the severity subgroups (i.e., the occurrence of an interaction with severity) might be accounted for by differences in the variance of the mental state scores across the severity subgroups. Furthermore, as linear regression analysis is not particularly suitable for the analysis of skewed data, we wished to investigate if a more appropriate method to analyse connection strengths, such as inverse Gaussian regression [10], would lead to different conclusions. Therefore, we asked Wigman et al. to share their data for reanalysis.

## Material and Methods

### Data

The data had been collected by Wigman et al. from 579 female general population siblings (aged between 18–61 years), sampled from the East Flanders Prospective Twin Study register [11]. In short, the women were classified into 4 severity subgroups based on the quartile scores of the SCL. The ESM was used to collect repeated measurements of mental states on 10 random moments during the day, on 5 consecutive days, thus providing a maximum of 50 measurement points per person. Women with less than 17 valid measurements were excluded by Wigman et al. Three mental states were measured: NA, PA, and PAR. For the measurement of NA the participants rated 5 adjectives (insecure, lonely, anxious, guilty, and down) on a 7-point Likert scale from 1 = “not at all” to 7 = “very”. PA was measured by rating 4 adjectives (happy, enthusiastic, energetic, and satisfied) and PAR was measured by rating a single item (I feel suspicious) on the same 7-point scale. The mental state scores were constructed by calculating the mean item score, so that all 3 mental state scores ranged from 1–7.

### Statistical analyses

#### Distributional characteristics

We started by examining the data using histograms and descriptive statistics. In addition, we examined the correlations between the mental state and severity variables, using both Pearson and Spearmen correlations because of the non-normality of some of the variables. We tried to redefine severity subgroups, based on the SCL-score in such a way that the subgroups would obtain equal variances.

#### Reanalysis using inverse Gaussian regression

The inverse Gaussian distribution is characterized by 2 parameters, a mean and a precision parameter [12]. When the “precision” is relatively low, the distribution is characterized by positive skewness and a long right tail, but when the “precision” is relatively high, the distribution approaches the normal distribution. All values of the inverse Gaussian distribution must be strictly positive (>0). As inverse Gaussian regression assumes a more or less positively skewed distribution, the method is particularly suitable for the analysis of symptoms in low-symptomatic populations. We used the package lme4 version 1.1–7 [13] as implemented in the statistical program R version 3.2.0 [14] to perform generalized linear mixed modelling with inverse Gaussian as response distribution and the log as link function. We included random intercepts and slopes, accounting for clustering of the measurements within persons within families. The variance-covariance structure of random effects was unstructured and we assumed an independent error-covariance structure at the lowest level. We used the original severity subgrouping, based on quartiles of the SCL-score, as categorical variable. The likelihood ratio test was used to test the significance of the mental state-severity interaction by comparing 2 nested models, one including severity as effect modifier, and one including severity as a covariate but without the mental state-severity interaction terms. Like Wigman et al. we regressed each of the mental states at time *t* on each of the mental states at time *t-1*. Note that, in this way, cross-lagged effects are not adjusted for autoregressive effects. Thus, if NA at time *t* is predicted by PAR at *t-1*, this may actually be due to the fact that NA at time *t* is predicted by NA at time *t-1* when NA and PAR at each time point are contemporaneously correlated (which is likely).

## Results

### Data description

**Fig 2:**
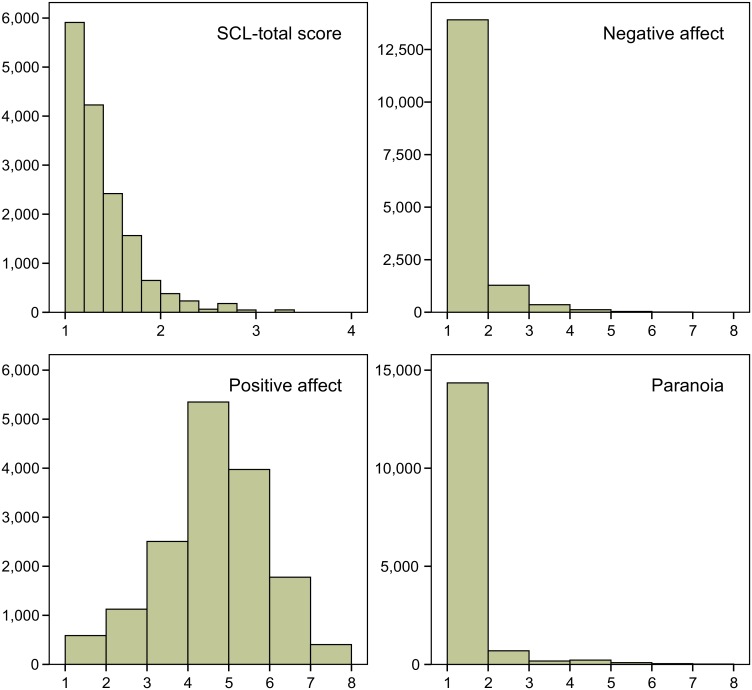
Distributions of the variables. Histograms of the variable scores’ distributions with the variable scores on the X-axis and frequencies of measurements on the Y-axis.

The data effectively contained 15,725 measurements of one or more mental states at time *t*, preceded by the measurement of one or more mental states at time *t-1* on the same day in 571 women with valid SCL-scores. Fig 2 displays the distributions of the SCL-score and the mental state scores (expressed as mean item scores). Note that PA had a fairly normal distribution. However, as we expected, NA, PAR and the SCL-score were significantly positively skewed.

Table 1 lists the descriptive parameters of the variables across all measurements for the total group as well as for the separate severity subgroups. We noted unexplained, but inconsequential, differences between the data and the standard deviations for NA, PA and PAR as reported by Wigman et al. on page 4. PA was reasonably normally distributed, whereas the SCL-score, NA and PAR were positively skewed—PAR to an extreme degree. Note that the skewness of NA and PAR was associated with considerable floor effects of these scales. Apparently, most women rated the NA and PAR adjectives with “not at all” most of the times. Even in de most severe subgroup 47.2% of the measurements of NA and 84% of the measurements of PAR indicated a “not at all” response to all items. Importantly, as we expected, the severity subgroups showed considerably different variances of the SCL-score, NA and PAR with the variance becoming larger with increasing severity levels. On the other hand, the variances of the (normally distributed) PA variable were more or less the same across the severity subgroups. The fact that Wigman et al. found evidence for staging when NA or PAR was the dependent variable, and not when PA was the dependent variable, could well be explained by the fact that the severity subgroups had different variances (increasing with severity) for NA and PAR, but not for PA.

**Table 1:** Distributional parameters of the variables by severity subgrouping.

Parameters	SCL-score	NA	PA	PAR
***Total group***				
** N (measurements)**	15,725	15,716	15,724	15,581
** Mean**	1.38	1.28	4.47	1.15
** Variance**	0.122	0.368	1.577	0.390
** Skewness**	1.93	3.17	-0.38	5.21
** Floor effect**^a^ **(%)**	0.5	68.2	1.2	92.1
** Ceiling effect**^b^ **(%)**	0.0	0.0	2.6	0.1
***Severity level 1 subgroup***				
** N (measurements)**	4,320	4,318	4,319	4,288
** Mean**	1.08	1.10	4.80	1.06
** Variance**	0.001	0.104	1.490	0.154
** Skewness**	-0.42	5.35	-0.59	8.95
** Floor effect**^a^ **(%)**	1.7	84.0	0.6	96.8
** Ceiling effect**^b^ **(%)**	0.0	0.0	3.3	0.1
***Severity level 2 subgroup***				
** N (measurements)**	3,908	3,907	3,908	3,873
** Mean**	1.20	1.18	4.48	1.10
** Variance**	0.002	0.180	1.398	0.229
** Skewness**	0.17	3.33	-0.40	6.34
** Floor effect**^a^ **(%)**	0.0	73.7	1.4	93.7
** Ceiling effect**^b^ **(%)**	0.0	0.0	2.5	0.0
***Severity level 3 subgroup***				
** N (measurements)**	3,659	3,659	3,659	3,632
** Mean**	1.40	1.27	4.43	1.13
** Variance**	0.005	0.304	1.513	0.324
** Skewness**	0.07	3.11	-0.35	5.39
** Floor effect**^a^ **(%)**	0.0	65.5	0.9	93.2
** Ceiling effect**^b^ **(%)**	0.0	0.0	2.0	0.0
***Severity level 4 subgroup***				
** N (measurements)**	3,838	3,832	3,838	3,788
** Mean**	1.87	1.60	4.12	1.33
** Variance**	0.124	0.771	1.675	0.843
** Skewness**	1.69	1.95	-0.17	3.37
** Floor effect**^a^ **(%)**	0.0	47.2	2.1	84.0
** Ceiling effect**^b^ **(%)**	0.0	0.0	2.4	0.3

NA = negative affect, PA = positive affect, PAR = paranoia

^a^ Floor effect: percentage of scores at the lower boundary of the scale

^b^ Ceiling effect: percentage of scores at the upper boundary of the scale

Table 2 demonstrates that the correlations between the variables were moderate. As expected, PA was negatively correlated with the other variables.

**Table 2:** Pearson product-moment and Spearman rank correlations of the variables.

	Pearson correlations			Spearman correlations		
	SCL	NA	PA	SCL	NA	PA
**Negative affect (NA)**	0.355			0.317		
**Positive affect (PA)**	-0.213	-0.360		-0.220	-0.345	
**Paranoia (PAR)**	0.174	0.453	-0.147	0.162	0.414	-0.128

All correlations were significant at p < 0.01

**Fig 3:**
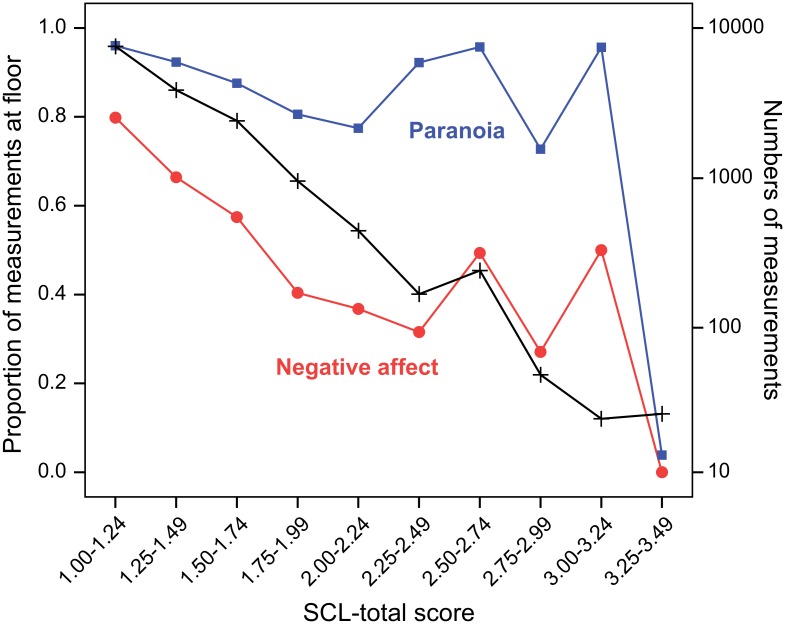
Floor effects of negative affect (NA) and paranoia (PAR) by SCL-total score level. Proportions of mental state (NA and PAR) scores at the floor of their scales by overall severity of psychopathology (SCL-total score). The red curve represents the proportions of floor scores for NA, whereas the blue line represents the same for PAR (scale on the left). Numbers of measurements by overall severity are displayed in the black line (scale on the right).

**Fig 4:**
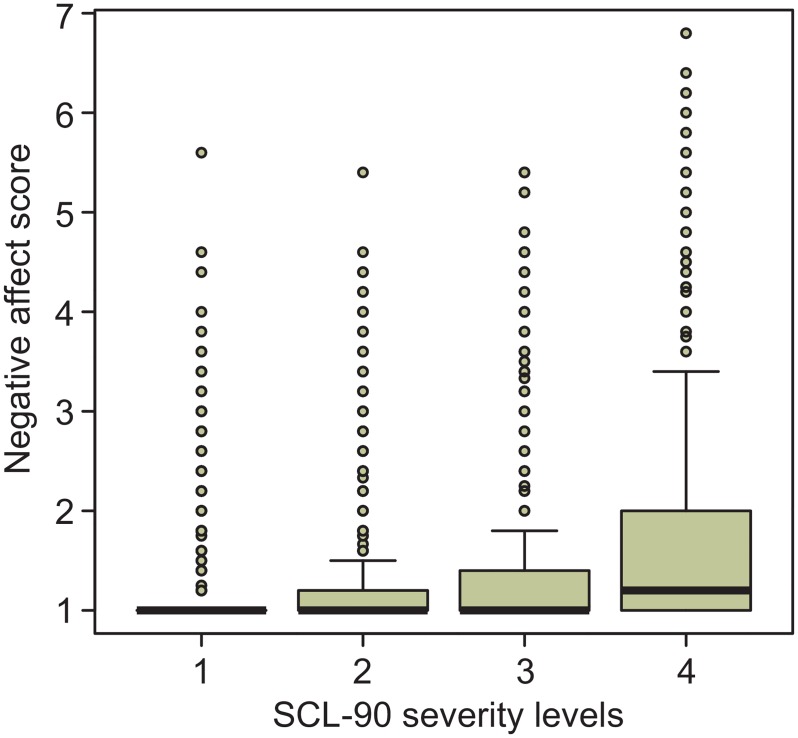
Boxplots of the distributions of the negative affect score across severity subgroups. The “boxes” indicate the interquartile ranges (IQR). The median observations are indicated by thick lines in the boxes. The “whiskers” extend to the highest (and lowest) observations not further away from the box than 1.5 times the IQR. Outliers are represented by small circles.

By shifting the cut-offs on the SCL-score we attempted to create new severity subgroups with (about) equal variances of NA and PAR. However, these attempts failed due to the fact that considerable proportions of measurements were at the floor of the NA and PAR scales, across all severity levels, except the highest SCL-score (Fig 3). Note, however, that the highest SCL-scores in Fig 3 concerned only two women with 24 and 26 measurements respectively. The number of measurements declined rapidly with increasing severity. So, irrespective of the exact locations of the cut-off points, in all groups considerable proportions of measurements remained at the floors of the scales, while as severity increased, increasing (but still relatively small) proportions of measurements reached higher scores, producing increasing variances (Fig 4). This pattern of having the greatest variance in the most severe group and the smallest variance in the least severe group remained the same irrespective of whichever way the cut-off points were shifting.

### Inverse Gaussian regression

The results of the inverse Gaussian regression models are presented in Table 3, which is comparable with Wigman et al.’s Table 2. Several models needed rescaling of the dependent or independent variable or both in order to obtain model convergence. If necessary, independent variables were rescaled by subtraction of the scale’s grand mean (over all persons and measurements), leaving the scale metric otherwise unchanged. Dependent variables were rescaled (if necessary) by adding 0.5 point, resulting in a scale range from 1.5–7.5 (instead of 1–7) and leaving the scale metric otherwise unchanged. Note that the original symptom scales (range 1–7) were basically completely arbitrary scales and that rescaling these scales did not principally affect the outcomes of the analyses, including the assessment of interaction effects. Table 3 displays subgroup-specific regression (B) coefficients and their 95% confidence intervals. As the link function was the log, these coefficients represent the change or difference in the natural logarithm of the dependent variable score associated with one unit change or difference in the independent variable. The exponent of this coefficient thus represents a score ratio, i.e., the ratio by which the dependent variable changed or differed associated with 1 unit change or difference in the independent variable. Consider, for instance, the regression coefficient of 0.049 for the effect of PAR at time *t-1* on the NA-score at time *t* in the lowest severity subgroup. As the exponent of 0.049 equals 1.05, a change or difference in PAR-score of 1 point at time *t-1* was associated with an expected NA-score change or difference at time *t* by +5%. Note that these effects reflect a mixture of between-person and within-person effects, but, following Wigman et al., we did not attempt to disentangle these effects. Similarly, in the highest severity subgroup, 1 point change or difference in PAR at time *t-1* was associated with a +8% (the exponent of 0.078 = 1.08) change or difference in NA-score at time *t*. Table 3 shows that most of the regression coefficients reached statistical significance at p < 0.05, which was largely comparable with the results of Wigman et al. However, most of the severity-mental state interactions (i.e., increasing effects of mental states at time *t-1* on mental states at time *t*) could not be confirmed. The only exception concerned the interaction between severity and PA with respect to the connection strength between PA at time *t-1* and NA at time *t*. In the successive severity subgroups, from the lowest to the highest, one point change or difference in PA at time *t-1* was associated with a change or difference in NA-score at time *t* by -1.9%, -3.8%, -4.5% and -8.1%, respectively (e.g., the exponent of -0.019 = 0.981, which indicates a change or difference by -1.9%).

**Table 3:** Mental states (negative affect, positive affect and paranoia) at moment *t* predicted by mental states at moment *t-1*, by SCL-severity. Method: inverse Gaussian regression analysis. Subgroup-specific regression (B) coefficients with 95% confidence intervals.

**Negative affect at moment *t* predicted by mental states at moment *t-1*, by SCL-severity**			
**SCL-severity**	**Negative affect at *t-1***	**Positive affect at *t-1*** ^a^	**Paranoia at *t-1***
**Symptom severity—level 1**	0.110 (0.059; 0.161)*	-0.019 (-0.037; -0.001)*	0.049 (-0.002; 0.100)
**Symptom severity—level 2**	0.175 (0.128; 0.222)*	-0.039 (-0.057; -0.021)*	0.088 (0.047; 0.129)*
**Symptom severity—level 3**	0.163 (0.119; 0.207)*	-0.046 (-0.064; -0.028)*	0.053 (0.016; 0.090)*
**Symptom severity—level 4**	0.163 (0.119; 0.207)*	-0.084 (-0.102; -0.066)*	0.078 (0.044; 0.112)*
**Significance of interaction with severity**	Chi-sq = 4.013; df = 3; p = 0.260	Chi-sq = 24.307; df = 3; p = 0.000	Chi-sq = 2.319; df = 3; p = 0.509
**Positive affect at moment *t* predicted by mental states at moment *t-1*, by SCL-severity**			
**SCL-severity**	**Negative affect at *t-1*** ^a^^b^	**Positive affect at *t-1*** ^a^	**Paranoia at *t-1*** ^a^^b^
**Symptom severity—level 1**	-0.066 (-0.107; -0.025)*	0.083 (0.067; 0.099)*	-0.017 (-0.054; 0.020)
**Symptom severity—level 2**	-0.075 (-0.109; -0.041)*	0.082 (0.066; 0.098)*	-0.036 (-0.067; -0.005)*
**Symptom severity—level 3**	-0.065 (-0.096; -0.034)*	0.084 (0.068; 0.100)*	-0.028 (-0.055; -0.001)*
**Symptom severity—level 4**	-0.072 (-0.103; -0.041)*	0.090 (0.075; 0.105)*	-0.030 (-0.054; -0.006)*
**Significance of interaction with severity**	Chi-sq = 0.229; df = 3; p = 0.973	Chi-sq = 0.731; df = 3; p = 0.866	Chi-sq = 0.605; df = 3; p = 0.895
**Paranoia at moment t predicted by mental states at moment t-1, by SCL-severity**			
**SCL-severity**	**Negative affect at *t-1*** ^b^	**Positive affect at *t-1*** ^b^	**Paranoia at *t-1*** ^b^
**Symptom severity—level 1**	-0.009 (-0.046; 0.028)	0.007 (-0.007; 0.021)	0.035 (-0.014; 0.084)
**Symptom severity—level 2**	0.047 (0.013; 0.081)*	-0.005 (-0.019; 0.009)	0.050 (0.010; 0.090)*
**Symptom severity—level 3**	0.046 (0.014; 0.078)*	-0.003 (-0.017; 0.011)	0.050 (0.014; 0.086)*
**Symptom severity—level 4**	0.017 (-0.017; 0.051)	-0.019 (-0.033; -0.005)*	0.026 (-0.008; 0.060)
**Significance of interaction with severity**	Chi-sq = 6.353; df = 3; p = 0.096	Chi-sq = 6.752; df = 3; p = 0.080	Chi-sq = 1.184; df = 3; p = 0.757

* p < 0.05

^**a**^ Independent variable needed rescaling (mean = 0)

^**b**^ Dependent variable needed rescaling (+0.5)

## Discussion

Several studies on networks have analysed and interpreted different connection strengths between mental symptoms in different (sub)groups. These connection strengths are often interpreted as indicative of the importance (“centrality”) of specific symptoms within their networks (e.g., [3,6]). Van de Leemput et al. interpreted the increase in symptom variance and (auto)correlation as signs of “critical slowing down”, a phenomenon suggestive of approaching a “tipping point”, i.e., a point in time where one (healthy) state transits into another (diseased) state, or vice versa [7]. The correctness of these interpretations depend on whether it can be demonstrated that the differences in connection strength are not solely due to differences in variance. It should be noted that the variance problem may also occur in single group studies where connections between symptoms are compared (e.g., [15]).

Wigman et al. interpreted increased connection strengths between symptoms as indicative of a hypothesized staging process, responsible for the development of mental syndromes from very mild, diffuse, non-specific conditions to severe fully developed mental disorders. Wigman et al. analysed connections between mental states in subgroups with different degrees of “range restriction” (due to the skewness of the severity measure). The effect of “range restriction” on measures of linear association, such as correlations and regression coefficients, is well known for a long time [4]. Differential range restriction (i.e., the variance in low severity groups is more restricted than in high severity groups) results in differential reduction in linear associations, thus in differential connection strength between symptoms. In other word, subgrouping based on a skewed severity distribution may well account for increasing connection strength between mental states.

Strictly speaking, we did not prove that the differential connection strength in the Wigman et al. data were solely due to differential variance reduction as we were not able to obtain subgroups with equal variances and we have not attempted to correct for the effect of differential range restriction (which is problematic with skewed data). Instead, we used an analytical method that is better suited for the analysis of skewed data (which constitutes the assumed source of differential variances and, thus, connection strengths). This alternative method failed to demonstrate increasing connection strength between mental states with increasing severity (i.e., with increasing variance) in all models (except one) indicating that, when the skewness of the dependent variable was taken into account, no differential connection strength could be discerned across the severity subgroups. We believe this to be an indirect, but compelling, argument for considering the differential connection strength in the linear analysis to be largely, artefactually, due to differential range restriction across the severity subgroups. True increasing connecting strength with increasing severity would have been revealed through inverse Gaussian regression.

One of the 9 models examined (i.e., the model in which PA predicted NA) showed increasing connection strength across severity subgroups while taking the skewed outcome variable into account. So, there might be minimal support for the staging hypothesis. However, although inverse Gaussian regression proved certainly to be less sensitive to differences in variance (in the other models), the method may not be entirely insensitive. Future studies (in particular simulation studies) should aim to clarify the extent to which differences in variance lead to differences in association measures using different analytical techniques, including inverse Gaussian regression. In addition, future research should focus on valid methods to control for differences in variance. As it currently stands, with that little support—possible support in 1 of 9 models—the verdict about staging should be that a process of staging (as evidenced by increasing connection strength between mental states with increasing severity) has not unequivocally been confirmed.

Finally, we would like to add some suggestions on how future research should investigate the development of psychopathology in general—and staging in particular. First, it is important to select study populations in which development of psychopathology can be expected to occur, such as stressed or traumatized populations. In relatively healthy populations, where symptoms show floor effects of 68% or more (as in the Wigman et al. data), lack of variability in scores constitutes a serious threat to any analysis of dynamic changes over time. Second, study populations should preferably be followed-up for periods long enough to actually observe the development of psychopathology, that is, at least several weeks or months. In current network studies, the actual development of psychopathology is often inferred from an assessment outside the period in which data are collected for mapping the networks. Network characteristics are then related to derived, but not observed, changes in psychopathology. The ESM paradigm may be an important hindrance because the collection of momentary measurements multiple (e.g., 10) times a day is difficult to sustain for more than a few days (5 days seems to be the norm). Third, the methodology to assess the strength of associations (connections) between network components (e.g., symptoms) needs to be further developed on order to deal with differences in variance. The fallacy of misinterpreting differences in associations between 2 symptoms as indicative of differences in the impact of one symptom on the other, while in fact the differences in associations are due to differences in variance, should be avoided.

## Conclusion

Reanalysis of the Wigman et al. data did not unequivocally confirm evidence of staging. Increasing connection strength between mental states may be largely due to differences in variances with increasing severity, and should not be interpreted as real phenomena.
